# Medical Items and News of Interest to the Physician

**Published:** 1871-10

**Authors:** 


### Of Interest to the Profession.

CULLED FROM THE STANDARD MEDICAL JOURNALS OF THE WORLD.

NOTE.—These formulæ are intended only for the regular practitioner of medicine, and should be employed only by him, or under his immediate supervision.

**Oakum.**

The use of oakum in surgery instead of lint, and which was highly appreciated by the German surgeons, in the recent war, is an American invention, the credit of which is due to Dr. Lewis A. Sayre, of New York.

**Condurango.**

This so-called cancer cure, which Dr. Bliss, of Washington, has so ingeniously advertised, proves to be worse than useless, as a specific for cancer. We have taken pains to ascertain the facts in the case and know whereof we speak.

**Cholera Infantum.**

Dr. Galloupe, of Lynn, Mass., (*Boston Med. and Surg. Jour.*), has employed, with good success, the following combination as diet in cases of cholera infantum: cream, one tablespoonful; water, four tablespoonfuls; lime water, one tablespoonful.

**Dysentery.**

Dr. Thos. J. Gallagher, of Pittsburg, Pa., (*N. Y. Med. Jour.*), says the pain and tenesmus accompanying dysentery is instantly relieved by from one to two hypodermic injections of morphia. One or two injections daily is all that is required to complete the cure.

**Anæmia.**

Dr. John Rose reports the case of an anæmic girl, aged 13 years, who had choria following rheumatism, who was successfully treated by ether spray, applied along the spine for four or five minutes each time; and after 15 sittings, a very marked improvement took place, followed by complete recovery.—*Med. News.*

**Varicose Ulcer of Leg.**

Prof. Andrews, of Chicago, (*Med. Examiner*,) recently had an obstinate case of varicose ulcer of the leg, of 15 years standing, which was cured by the injection of tinct. of ferri murias into the varicose veins, and a dressing of an antiseptic ointment, composed of carbolic acid cyst., 18 grs.; adipis. one drachm.

**Puerperal Convulsions.**

Dr. J. W. Richardson, of Smyrna, Rutherford Co, Tenn., has been appointed by the State Medical Society, to prepare a paper on puerperal convulsions. He wishes to collect statistics, and will send a blank form with inquiries to any physician who will respond to him.

**Prevention of Pitting in Small-Pox.**

The Melia Azidavaclita L. of India is used in that country by the natives to cover the bodies of patients recovering from small-pox, it being believed to prevent the marks becoming permanent. Dr. Wight says of it: The leaves beaten into a pulp and externally applied, act like a charm in removing the most intractable form of psora and other pustules of an eruptive nature.

**Hiccough Cured by Chloral.**

Dr. P. F. Whitehead has recently prescribed for a patient with hiccough, which had continued for 36 hours. Various remedies where used with but little good effect, save a temporary cessation by the use of morphine hypodermically. Thirty grains of chloral hydrate gave immediate and permanent relief.—*Lancet.*

**Epithelioma Removed by Bromine.**

Dr. Wynn Williams showed to the Obstetrical Society of London, a patient nearly the whole of whose lower lip had been removed for epithelioma eighteen months previously. The disease shortly appearing in the cicatrix, the growth was successfully treated by two injections of bromine, twenty drops to a drachm of spirit. There was no appearance of the disease at present.—*Lancet.*

**Chloral Hydrate and Cod-Liver Oil.**

The *Gaz. Farm. Ital.*, (*Medical Times and Gazette*,) advocates the addition of chloral hydrate to cod-liver oil. It renders it much less nauseous, and prevents the night-sweats of the phthisical patient, induces sleep, and creates appetite. The pure chloral hydrate crystals may be added to cod-liver oil in the proportion of ten grains of the former to one hundred and ninety of the latter.

**Incontinence of Urine.**

John Barclay, M. D., of Aberdeen, (*Med. Times and Gazette*), during the past two years and a half has successfully treated twenty cases of incontinence of urine by the syrup of the iodide of iron alone. The improvement in several of the cases followed so closely on the administration of the remedy as to leave no doubt but that the good effect was due to the syrup. Dr. Manson, and Dr. Smith of Kinnairdy, have both found the medicine equally satisfactory.

---

**Hæmoptysis.**

Dr. E. Holden, of Newark, N. J., writes to the *Record* that he has succeeded, frequently, in arresting profuse hœmorrhage by throwing the atomized vapor of a saturated solution of gallic acid, directly into the mouth and throat.

He says, "Unlike other styptics thus administered, it quiets the spasmodic cough, which seems the direct result of the presence of the blood, requires but a moment to prepare, and, aside from its efficacy, it inspires immediately the confidence of the patient."

---

**Secondary Lymph.**

Dr. J. B. Barbour, (*Lancet*,) Physician and Medical Superintendent of the Metropolitan Fever Hospital, Stockwell, says, "It is in the highest degree probable that re-vaccination by secondary lymph—that is, lymph taken from a vesicle produced by a secondary vaccination--does *not* protect from small-pox."

---

**How is This?**

Surgeon H. F. Patterson, of the Royal Artillery, (*N. Y. Med. Jour.*,) writes to the *Lancet*, that he has for some time been in the habit of treating gonorrhœa with water only. He begins with injections of luke warm water, and continues them hourly until chordee and scalding cease, and then uses cold water in same way, until the case is cured. He uses no internal treatment, unless an occasional saline aperient, and says he has not had a single failure.

---

**Otorrhœa.**

Dr. F. E. Webber, (*Berlin Klin. Woch.*,) states that spirit of wine is the best topical application in cases of otorrhœa uncomplicated with caries or polypus growth. The ear must first be well syringed out; then, the patient lying down, as much of the pure spirit as the ear will contain, is poured in and allowed to remain five minutes. After the spirit flows out, the meatus must be dried and plugged so as to prevent the access of air. At first it is to be applied three times a day, then twice, and is to be continued for some time after the otorrhœa has ceased.

**Intermittent Fever.**

Dr. Preulich, a German physician of note, reports eight cases of intermittent fever promptly cured by carbolic acid. His formula is:—

℞
Acidi carbolici, gr. iij;
Inf. gent., ℥ v;
Syrp. simpl., ℥ j. M.

Dose, ʒ j. ter die.

He explains that this successful use of carbolic acid proves that the action of quinine in intermittent fever is antiparasitic.—*From Wein Med. Presse, in Boston Med. and Surg. Journal.*

---

**Typhoid Fever.**

Dr. John E. Owens, Chicago, (*Chicago Med. Jour.*), observes, that during the last four years, both in hospital and private practice, milk and the acid and strychina mixture have been administered to patients with typhoid fever with success. The mixture is prepared as follows:

℞
Acid sulph. aromat., ʒ iij;
Strychinæ sulph., gr. 1-73;
Syrup. simpli., ℥ v. M.

DOSE.—A tablespoonful.

There is one noticeable feature in cases treated by Strychnia, viz: the dry, brown tongue soon becomes moist, and remains so during the treatment; this is effected, he believes, mainly through the agency of strychnia, by increasing the nutrative assimilative functions of the system.

---

**Turpentine in Headache.**

Dr. Warburton Begbie, in the *Edinburgh Medical Journal*, recommends the use of turpentine in the severe headache which is apt to occur in nervous and hysterial women. "There is," he says, "another class of sufferers from headache, and this is composed of both sexes, who may be relieved by turpentine. I refer to the frontal headache, which is most apt to occur after prolonged mental effort, but may likewise be induced by unduly-sustained physical exertion,—what may be styled the headache of a fatigued brain. A cup of very strong tea often relieves this form of headache; but this remedy, with not a few, is perilous, for, bringing relief to pain, it may produce general restlessness and—worst of all—banish sleep. Turpentine, in doses of twenty or thirty minams, given at intervals of an hour or two, will not only remove the headache, but produce, in a wonderful manner, that soothing influence to which reference has already been made."

**Iodoform Ointment.**

In the Boston City Hospital, iodoform ointment in connection with iodide of potassium is extensively and successfully used in the treatment of syphilitic ulcers and rupia. Dr. William Ingalls, attending surgeon, advocates this formula in two obstinate cases under his care.

℞.

Iodoformi, ʒ ss.

Spts. vini. rect., q. s.

Adipis suill, ʒ vijss. M.

—*Boston Med. & Surg. Journal.*

---

**Senile Gangrene.**

Dr. J. C. Butcher, North Lewisburg, Ohio, (*Leavenworth Medical Herald,*) publishes a case of senile gangrene, in which the administration of chloral hydrate had a magical effect in giving rest to the patient, hypodermic injections of morphia failed to give relief. He finds that small doses of chloral produce the same effect as large ones. His formula is as follows:—

℞

Chloral, ʒ j;

Simple syrup, ℥ j. M.

Dose, a teaspoonful every hour.

---

**Cathartic Pill.**

A writer in the *Chicago Med. Jour.* suggests the following formula, which he would substitute for the ordinary compound cathartic pill:

℞ Aqueous extract of aloes, podophyllin, gamboge, āā gr. xxx; oil of caraway, ♏ vj. M. Fiat pil. no. lx. He has had the pills made up and sugar-coated in a very satisfactory manner by J. W. Ehrman, a Chicago pharmaceutist. The pill is small, weighing only three grains after being sugar-coated. One pill is sufficient for a cathartic, without occasioning either pain or nausea.

---

**Rheumatism.**

Dr. R. H. Boyd states that he cures inflammatory rheumatism in from three to seven days by the following method: He gives first a full emetic dose of ant. et potass. tart., and when this has operated, five drops of tinct. opii and five drops tinct. colchici, every three or four hours; and a teaspoonful of a half-pint mixture, containing ʒ iv. potass. acet. every hour. When the patient becomes very hungry, and is quite free from pain, having fasted several days, he allows two tablespoonfuls of milk or one oyster, three times a day, increasing the quantity gradually each day.—*Michigan University Med. Jour.*

**Nephrotomy.**

Dr. Meadows, (*British Medical Jour.*,) performed the operation of nephrotomy July 1st, under peculiar circumstances. The operation was intended for the relief of a patient supposed to be suffering from the presence of an ovarian cyst, but, upon opening the abdomen, the tumor was found to be a large cyst of the kidney. The true structure of the organ had almost entirely disappeared, while the opposite kidney was apparently healthy. Dr. Meadows, in the belief that the removal of the diseased organ presented the best method of treatment, accordingly applied a ligature, as in the operation for ovariotomy. The case proved fatal on the sixth day, from hemorrhage from the pedicle.

---

**Carbolic Cerate.**

The following will be found to be an excellent formula for this preparation:

℞.

Adipis, ℥ x;

Ceræ albæ, ℥ v;

Terebinth. can., ℥ j;

Acid. Carbol, ℥ j.

Melt the lard and wax together, add the balsam fir, and when it begins to cool, stir in the carbolic acid.

The addition of balsam fir to this preparation corrects the disagreeable odor of the acid, and renders it slightly adhesive, which is quite desirable when the compound is used as a dressing for burns, old sores, &c.

---

**Severe Burns.**

Dr. Baker, of Andover, N. Y., details a case in *Med. and Surg. Reporter*, of severe scalding of a little child who fell into a tub of scalding water. The case recovered rapidly under the following treatment:—

℞
Beeswax, melted and strained, ℥ j;
Flaxseed or sweet oil, f. ℥ ij;
Tannic acid, ʒ j.

Mix as follows: Put the wax in a clean tin vessel and melt it; add the oil, stirring until thoroughly incorporated with each other; allow it gradually to cool while continually stirring it, then add the tannin. When cold, add six or eight drops of carbolic acid to the ounce of the mixture. Spread the mixture on strips of cloth and apply to the denuded surfaces, securing them with a roller bandage, loosely applied. The application may be renewed every three or four days.

**Croup.**

Dr. I. B. Kieffer, of Carlisle, Pa., writing to the *Chicago Med. Journal*, extols the following formula, for the treatment of croup:—

℞.
Bromide of potassium, gr. xx;
Chlorate of potassa, gr. x;
Ipicac, gr. j;
Ext. liquorice, ʒ ss;
Water, f. ℥ ijss. M.

Sig:—A teaspoonful every hour. Dr. W. W. Dale, (*Med. Times*), who has been using the same treatment, informs us that membraneous croup, spasmodic croup, and laryngitis, alike have lost for him nearly all that dread and anxiety with which he once met them.

**Chorea.**

In a severe case of chorea, reported by Dr. Hayden, (*British Med. Jour.*,) the patient, a girl, twelve years of age, took the one-twentieth of a grain of strychnia, in mixture, three times a day. This treatment was continued until slight rigidity of the muscles of the back of the neck was observable, with the effect of moderating the contortions of the shoulders and trunk. She was afterwards put on half-drachm doses of the syrup of the triple phosphate (strychnia, quinia and iron) three times a day. This produced the most beneficial results. A recovery was effected in about six weeks,—not a long time, when the severity of the case is taken into consideration.

**Treatment of Gonorrhœa.**

Mr. Berkley Hill (*Lancet*), speaks favorably of the jelly of copabia in the treatment of the sub-acute form of this disease. This preparation is almost as firm as calf's-foot jelly, very attractive to the eye by its rosy red color, and not repulsive to the palate, its flavor being masked with peppermint. As it contains seventy-five per cent. of copabia, large quantities can be taken in small bulk. If a piece as large as a filbert be rolled in a wafer paper, it can be swallowed without being tasted at all. The after-effects of nausea, diarrhœa, etc., are not more frequent than from other forms of copabia.—The following formula of Henderson is recommended in conditions not permitting the use of copaiba:—

℞
Oil of sandal-wood, f. ℥ j;
Rectified spts. of wine, f. ℥ ij;
Oil of cinnamon, ♏. xxv. M.

Sig:—Take one or two drachms three times a day.

**Summer Complaints of Children.**

Dr. F. G. Williams, of Waterloo, Wis., writes to the *Chicago Med. Examiner* that he has as much confidence in the use of bromide of potassium in the summer complaints of children, as Dr. Caro, of New York, who has long advocated its use.

At first Dr. W. used the bromide alone, in simple solution, gr. xx to gr. xxx, to the ℥ j.—Now he is in the habit of using syrp. rhubarb as a menstruum:

℞.
Syrp. Rhubarb, ℥ ij;
Pot. brom., ℈ ij. M.

Sig:—gtt. x to xx every one, two or three hours. If there is much pain or restlessness, from one-half to one grain morph. sulph. is added.

One of the most reliable stimulants is this:

℞.
Hoffman's Anodyn.
Paregoric,
Tinct. Camph, āā ℥ ij. M.

DOSE.—From ʒ j to ʒ iij, moderately diluted with water.

**Bullet in the Brain Nineteen and a Half Years.**

The *Journal of Psychological Medicine* contains the following translation from an Italian journal: "Joseph Soler, a lawyer in Venice, was shot in a duel, June 16, 1850. The ball entered the head above the right ear. Prof. Corteri saw the wound five hours afterwards. On 31st of Aug. he was deemed convalescent. Memory weak for some time, and vision disturbed. Soon the symptoms disappeared; mind became clear and judgment good as ever. Complained of pain in back and lower extremities, especially in the night, which became more acute in coughing or sneezing. Died of pneumonia Dec. 7, 1869. The autopsy displayed a funnel-shaped cicatrix above the right ear, and a notable thickening of the skull. On the petrous portion of temporal bone, and on border of tentorium, a dark body was found, which consisted of two pieces of lead, separated from each other by a splinter of bone. On rising the cerebellum, the finger could be thrust into a canal, which was in the brain-substance. At the end of this canal, which was ten centimetres in length and ran horizontally, another rough body was found, which proved to be a piece of bone two centimetres in length."

**Treatment of Croup by Inhalation of Glycerine.**

A German physician, Dr. Stehverger, recommends the treatment of croup by the inhalation of pure glycerine through one or other of the well-known forms of atomizing apparatus. He was led to try this remedy for croup from observing its good effects in cases of hoarseness and loss of voice. After application, the cough becomes more free and moist, and children are enabled to sleep almost immediately upon being relieved by the inhalation. It is, however, believed to be of importance to make use of the remedy early and frequently, as, if delayed, it may have no effect whatever. If the glycerine be pure, it may be used unmixed; if not, it should be diluted with a little water. The inhalations are repeated, according to the necessity of the case, at intervals of from half an hour to an hour and a half, and for about fifteen minutes at a time. The effect of the glycerine in this case is supposed to be due to the fact that the secretions of the mucous membrane are thereby increased, and tumefaction reduced.

---

**Epilepsy.**

B. W. Stone, M. D., Asst. Physician to the Western Lunatic Asylum, Kentucky, (*Richmond and Louisville Med. Jour.*), remarks that, although he has not effected any cures with bromide of potassium in epilepsy, the efficiency of this drug in prolonging, in some cases indefinitely, the interval between the attacks, in often warding off immediately threatening attacks, in procuring sleep and relief from the terrible cephalagia so often preceding or succeeding convulsions, in moderating the convulsive movements (during sleep), in shortening their duration, certainly renders it an invaluable boon to the epileptic. In the selection of a prescrpition for general use, hê refers to the experienced judgment of Brown-Sequard, who combines with the bromide the iodide of potassium, the bicarbonate of potassa, and the bromide of ammonium, in about the following proportions:—

℞

Potassæ, bicarbonatis, ℨ ij;

Ammonii bromidi, ℨ vij;

Potassii iodidi, ℨ iij;

Potassii bromidi, ℥ iij;

Infus. calumbæ, (British), O j. M.

Give a teaspoonful before each meal, and three teaspoonfuls at bed time, to be taken in water.

**Diphtheria.**

Dr. Jas. E. Reeves, Wheeling, W. Va., (*The Med. Times,*) has abandoned the use of strong caustic or astringents to the throat in the treatment of diphtheria. He uses gargles or the atomized spray of the chlorine and iron mixture, the following being his formula:—

℞

Pot. chlorat., ℨ ij;

Acid. hydrochloric puri, ℨ iss;

Aquæ, f. ℥ viij;

Tinct. ferri chl., f. ℥ j. M.

Use with the atomizer or as a gargle, every hour when the patient is awake. At the same time the mixture may be internally administered, in doses of twenty drops to a teaspoonful, with or without syrup, every two or three hours. Warm atomized inhalations of the above mixture in diphtheritic croup is of the greatest advantage.

---

**Pertussis.**

Dr. John J. Caldwell, of Brooklyn, contributes to the *Boston Med. and Surg. Journal* the description of a method of treating whooping-cough by the application of atomized fluids to the air-passages, in accordance with the theory of Niemeyer, that the disease is a catarrh of the respiratory mucus membrane, attended with intense hyperæsthesia. The result of this treatment, in his own practice, has been that the whooping inspirations have ceased within a week, and the catarrhal inflammation has rapidly subsided. The following is his formula:—

Ext. belladonnæ, fl. gtt. v ad x;

Potassæ bromidi, ℈ j;

Ammoniæ, bromidi, ℈ ij;

Aquæ distill., f. ℥ ij.

Fiat solutio.

Of this, apply a teaspoonful once daily, by means of the atomizer.

---

**Measles.**

Dr. N. S. Davis, of Chicago, (*Med. Examiner*), considers the following formula one of the best preparations in the first stage of severe cases of measles:—

℞
Syrup scillæ comp., f. ℥ jss;
Vinum antimonii, f. ℥ ss;
Tinct. opii camph., f. ℥ ij;
Tinct. veratrum viride, ℨ j. M.

Dose, one teaspoonful every three hours in a tablespoonful of water. This will, usually, in the course of 24 hours lessen the fever and modify the cough, while the pain in the head will at the same time be greatly relieved. He is not in the habit of giving cathartics until the eruption is fairly out; when, if the bowels have not moved for a couple of days, he directs a mild laxative like the following:

℞

Hydrarg. chl. mit. gr. v;

Leptandrin, gr. ij;

Sodæ bicarb., gr. v. M.

One important thing to guard against is, extension of the irritation from the bronchial tubes to the lobules of the lungs; making it complicated with lobular pneumonia. When symptoms of pneumonia occur in connection with measles, the best remedy in children is a combination like the following:—

℞
Liq. ammon. acetas, ℥ iss;
Syrup ipecac., ℥ ss;
Tinct. opii camph., ℥ j;
Tinct. veratrum viride, ʒ j. M.

Dose—proportioned to the age of the child; for a child two years old, about twenty drops; for an adult, one teaspoonful. It should be given every two, three, or four hours, till the fever is controlled. In the active stage of the disease, while using this mixture, cover the chest externally with fomentations.

Dr. Davis has considerable faith in the popular notion about onions; they certainly afford more relief in the breathing than any other thing. He attributes it to the impregnation of the air which is inhaled, with the volatile oil, more than to any absorption from the surface of the chest, and thinks this application preferable to blistering.

---

**Acute Dysentery.**

Dr. N. S. Davis, (*Chicago Med. Examiner*), recommends the subjoined treatment in acute dysentery:—

℞.
Ol. terebinth.,
Tinct. opii,
Acacia,
Sacch. alb.,
Aqua menth., āā ℥ iij. M.

One teaspoonful every four hours. And a solution of carbolic acid, as follows:

℞.
Carbolic acid, cryst., gr. vj:
Tinct. opii camphor., ℥ i ss;
Glycerine, ℥ ss;
Water, ℥ ij. M.

One teaspoonful every four hours, alternating with the emulsion.

